# The Effect of Shiatsu Massage on Underlying Anxiety in Burn Patients

**Published:** 2015-01

**Authors:** Fatemeh Mohaddes Ardabili, Soybeh Purhajari, Tahereh Najafi Ghzeljeh, Hamid Haghani

**Affiliations:** 1 Medical Surgical Group, School of Nursing and Midwifery, Iran University of Medical Sciences, Tehran, Iran;; 2 Statistic and Mathematics Department, School of Health Management and Information Sciences, Iran University of Medical Sciences, Tehran, Iran

**Keywords:** Anxiety, Burn, Shiatsu massage, Pain

## Abstract

**BACKGROUND:**

Burn patients experience high levels of predictable anxiety during dressing changes while anti-anxiety drugs cannot control these anxieties. The nurses can limit the side effects of medications by undertaking complementary therapies. Hand pressure massage was introduced as a technique that can reduce these anxieties. This study aimed to investigate the effect of hand pressure massage using Shiatsu method on underlying anxiety in burn patients.

**METHODS:**

In an available randomized study, 60 burn patients with underlying pain were enrolled. They were randomly allocated in two groups of hand massage and the control. The anxiety of underlying burn pain before and after the massage was evaluated using Burn Specific Pain Anxiety Scale (BSPAS).

**RESULTS:**

The difference for anxiety scores in the hand Shiatsu massage group before and after massage were statistically significant, but in the control group was not significant.

**CONCLUSION:**

Based on our findings, 20 minutes of hand Shiatsu massage in conjunction with analgesic medications can be beneficial to control the anxiety of burn patients.

## INTRODUCTION

Burn is still one of the devastating conditions in emergency medicine affecting all age groups and both genders that can lead to physical, psychological and chronic disabilities.^1^ During pregnancy, it is an emergency event due to its increasing trend in mortality and morbidity of both mother and infant.^2^ Due to undertaken suicides, education seem necessary to decrease this awful event and its physical and emotional complications.^3^ Silver sulfadiazine was introduced as the gold standard in topical burn therapy with antibacterial properties,^4^ even medicinal plants were introduced in wound healing of burned injuries.^5^^-^^8^

Burn patients always suffer from the anxiety of treatment following burns.^9^ They may experience high levels of predictable anxiety during dressing changes, and the anxiety increases after dressing change and over time by treatment measures while the anti-anxiety drugs cannot control it.^10^ Restlessness, sadness, loss of appetite, increase of blood pressure, irregular breathing, and palpitations are the most important symptoms of anxiety. These factors in burn patients which their immune system is weak can cause extensive damage. Thus, anxieties of uncontrolled origin can be harmful effecting the health of burn patients.^11^

There is a possibility to reduce the anxiety with various complementary sedative measures.^12^ Directly, nurses are in the front line who are practicing these techniques.^13^ They can apply complementary therapies to limit the side effects of medications.^14^ The relaxation techniques are the most important non-pharmacological methods which are used to control anxiety. Non-pharmacological approaches are affected through the hypothalamus on the parasympathetic nervous system, causing a decrease in the heart rate, blood pressure, metabolism, respiration rate and oxygen consumption.^15^ There are many ways to achieve relaxation such as massage therapy as one of the relaxation techniques that can improve relaxation and reduce the anxiety.^16^ An appropriate and desirable massage can provide the security and intimacy, also can reduce the anxiety. The massage can improve the communication between the nurse and the patient.^17^ Hand massage is one of those that was introduced as a technique that can reduce anxiety.^18^ This study aimed to investigate the effect of Shiatsu method of hand pressure massage on underlying anxiety in burn patients.

## MATERIALS AND METHODS

During 2013, in a randomized trial using available sampling method, 60 burn patients admitted to Shahid Motahhari Hospital in Tehran, Iran with an underlying anxiety were enrolled. The patients were randomly allocated in two groups of hand massage and control. The patients suffered from 10 to 45 percent of burn injuries. Healthy areas were available on the hands for the massage (at least from fingertips to elbow). Massage was performed for 20 minutes (10 minutes for each hand). The anxiety of burn pain before and after the massage was evaluated using Burn Specific Pain Anxiety Scale (BSPAS). The data were analyzed by SPSS software (Version 17, Chicago, IL, USA) using independent t-test. A *p* value less than 0.05 was statistically considered significant.

## RESULTS

**Fig. 1:**
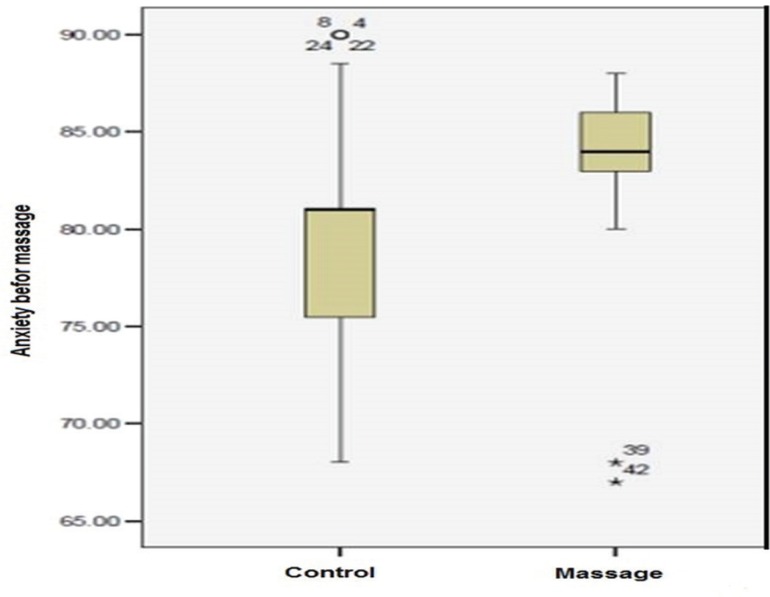
Anxiety score in the control and massage groups before intervention

**Fig. 2:**
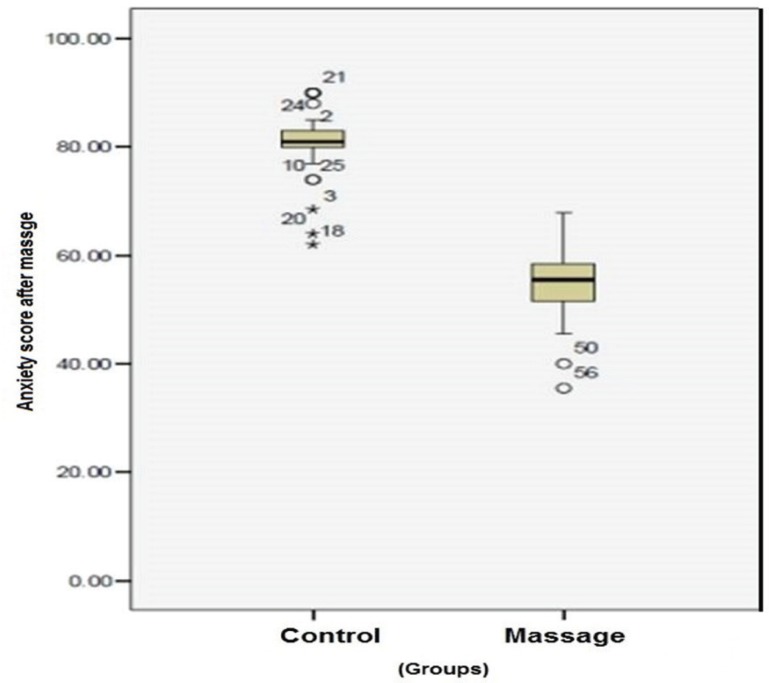
Anxiety score in the control and massage groups after intervention

The paired t-test findings showed that scores of pain anxiety before and after intervention in the control group was not statistically significant (P=0.89). The anxiety scores in the massage group before and after massage were statistically significant (P=0.001). The anxiety which was 83.03±4.69 before the intervention reduced immediately after intervention (55.02±7.09) (Table 1, Figures 1 and 2).

**Table 1:** The scores of underlying anxiety before and after intervention, in both groups

**Groups**	**Hand massage**	**Control**
**Underlying Anxiety**	**Mean±SD**	**Mean±SD**
Before massage	4.69±83.03	5.79±80.33
After massage	7.09±55.02	6.75±80.50
Difference	8.25±28.03	7.02 -17
Results of T-test	T=17.86, Df=29, P=0.001	T=0.13, DF=29, P=0.89

## DISCUSSION

Our findings showed that there was a statistically significant difference (P=0.001) for underlying anxiety scores before and after hand massage. The anxiety which was 83.03±4.69 before the intervention reduced immediately after intervention (55.02±7.09). So, Shiatsu massage for duration of 20 minutes on the hands could reduce the anxiety of burn patients. In our study, the anxiety scores increased before intervention (80.33±5.79) when compared to after the intervention (80.50±6.75). In the control group, the difference for anxiety (without massage) before and after trail was not statistically significant. 

A significant difference (P=0.007) was demonstrated for anxiety before and after the pressure massage in patients with a generalizing anxiety disorder.^19^ It was shown that hand massage could reduce the anxiety in patients waiting for surgery. They received massage and reported experiencing lower levels of anxiety in comparison to those who received a common care by nurses.^20^ The reports revealed that nurses can independently decrease the pain anxiety in burn patients and the subsequent physical and psychological burden by educating them with a simple and inexpensive technique of jaw relaxation.^21^ The massage can affect neurotransmitters in the brain, increase the serotonin and dopamine levels that can help in reduction of anxiety. So, the massage can reduce the heart rate, blood pressure, metabolism, respiratory rate and the oxygen consumption.^22^ Also, the hand pressure massage performed by lowering of the muscle strain resulted into the relaxation and reduction of anxiety.^23^ Reduction of pain, anxiety, respiratory rate and diastolic blood pressure were previously reported after hand massage.^24^^,^^25^ We can conclude that based on our findings, 20 minutes of hand massage in conjunction with analgesic medications could control the anxiety of burn patients.

## Note

Please cite this paper as:

Mohaddes Ardabili F, Purhajari S, Najafi Ghzeljeh T, Haghani H. The Effect of Shiatsu Massage on Underlying Anxiety in Burn Patients. World J Plast Surg 2015;4(1):36-39.
